# Comparing Tibiofemoral Rotation Measurements Between Computed Tomography and Magnetic Resonance Imaging in Patients With Patellofemoral Instability

**DOI:** 10.1177/23259671241304754

**Published:** 2025-01-15

**Authors:** Lukas Jud, Alexander Berger, Martin Hartmann, Lazaros Vlachopoulos, Jakob Ackermann, Sandro F. Fucentese

**Affiliations:** † Department of Orthopaedics, Balgrist University Hospital, University of Zurich, Zurich, Switzerland; Investigation performed at Balgrist University Hospital, Department of Orthopaedics, University of Zurich, Zurich, Switzerland

**Keywords:** anterior cruciate ligament, knee, knee rotation, knee version, osteotomy, patella, patellofemoral instability, trochlear dysplasia

## Abstract

**Background:**

Tibiofemoral rotation is an emerging parameter, especially in assessing patellofemoral instability. However, reference values in the literature are inconsistent regarding the used imaging modality and do not consider the effect of knee flexion during image acquisition.

**Purpose:**

To analyze the differences in tibiofemoral rotation measurements between computed tomography (CT) and magnetic resonance imaging (MRI).

**Study Design:**

Cross-sectional study; Level of evidence, 3.

**Methods:**

A total of 78 knees in 72 patients were included. All patients underwent surgery for patellofemoral instability at our institution and preoperative CT and MRI were available. Tibiofemoral rotation was measured on axial CT and MRI, whereas the respective knee flexion angle (KFA) was measured on sagittal images. Tibiofemoral rotation values in which the tibia was externally rotated to the femur were handled as positive values. Differences between CT and MRI measurements were calculated and the association between KFA and tibiofemoral rotation was evaluated using Pearson correlation and the Mann-Whitney *U* test.

**Results:**

The mean tibiofemoral rotation was 8.7°± 5.5° in CT and 4.2°± 6.7° in MRI (*P* < .001). The mean KFA was 2.4°± 3.1° in CT and 14.9°± 6.4° in MRI (*P* < .001). The difference in the KFA between CT and MRI moderately correlated with the difference in tibiofemoral rotation between imaging modalities (*r* = 0.529; *P* < .001).

**Conclusion:**

Tibiofemoral rotation measurements significantly differed between CT and MRI, with larger values observed in CT. The difference between imaging modalities correlated with the degree of knee flexion during image acquisition. This observation should be considered when assessing tibiofemoral rotation, as current reference values in the literature are inconsistent regarding the used imaging modality.

Patellofemoral instability is a multifaceted pathology, which requires a comprehensive assessment to identify the origin of the instability and to plan the respective treatment. The underlying contributing anatomical factors include trochlear dysplasia, torsional deformities of the femur and tibia, frontal malalignment of the leg, patellar height, or increased tibial tuberosity-trochlear groove (TT-TG) distance.^8,10,13,18,22^ The relative rotation of the femur on the tibia—called the tibiofemoral rotation—is a parameter that has received increasing attention in recent years. A correlation between anterior knee pain and increased values of tibiofemoral rotation was already demonstrated in the first description^9^ in 1997. Within the last year, different studies have shown a correlation between increased values of tibiofemoral rotation and patellofemoral instability.^4,12,16,21^ Recently, an increased tibiofemoral rotation was even identified as a risk factor for graft failure in anterior cruciate ligament (ACL) reconstruction.^15^ However, as the tibiofemoral rotation is measured between the tangent of the posterior femoral condyles and the tangent of the posterior tibial plateau condyles, it has to be considered that this parameter depends on the degree of knee flexion. The same characteristic was already observed for TT-TG distance measurements.^3,5,7,17^ Therefore, measurements of tibiofemoral rotation using computed tomography (CT) or magnetic resonance imaging (MRI) should be carefully differentiated, as the knee is normally in full extension in CT and in slight flexion in MRI because of the required MRI knee coils.^1^ The literature on tibiofemoral rotation has so far been inconsistent regarding the use of CT and MRI, with reference values for patellofemoral instability ranging from 6.9° in MRI to 12° in CT.^4,21^ To our knowledge, no study has yet examined the difference between CT and MRI in measuring tibiofemoral rotation. Therefore, this study aimed to evaluate the difference in tibiofemoral rotation measurements using CT and MRI data of patients with patellofemoral instability. We hypothesized that tibiofemoral rotation would be greater in CT data than in MRI data because of the screw-home mechanism with the knee in full extension.

## Methods

Our local ethical committee approved this study (Zurich Cantonal Ethics Commission, BASEC-Nr. 2021-01428) and all patients gave their informed consent.

A descriptive laboratory study was performed. All skeletally mature patients who underwent surgery for patellofemoral instability between January 2019 and July 2023 at our institution were identified. The inclusion criteria consisted of preoperative available standardized CT of the lower extremity with the knee in full extension via a specifically developed protocol per the regions of interest (ie, the hip, knee, and ankle joints) and available MRI of the knee. Patients with any type of previous surgical intervention of the affected knee were excluded.

### Radiological Assessment

**Figure 1.**
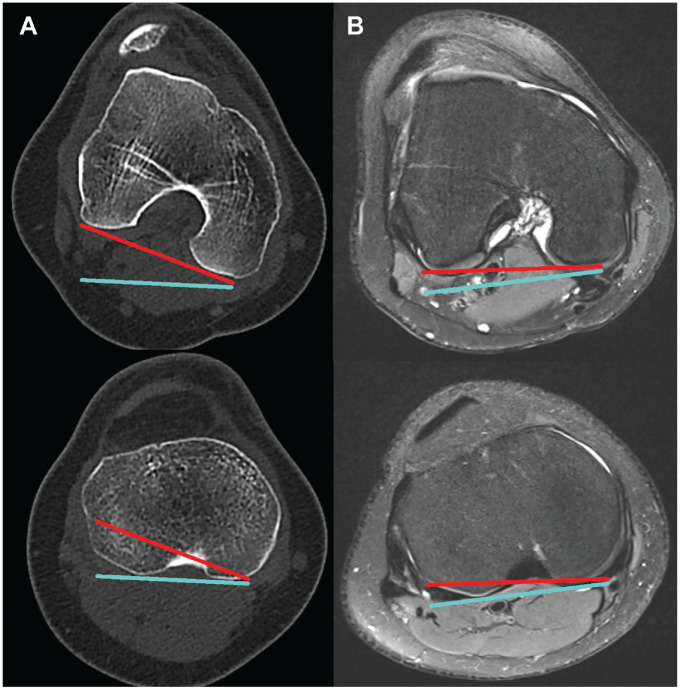
Measurement of tibiofemoral rotation in (A) CT and (B) MRI. The tangent line on the posterior femoral condyles is visualized in red. The tangent line on the posterior tibial condyles is visualized in turquoise. The tibiofemoral rotation is measured as the angle between the red and turquoise lines. CT, computed tomography; MRI, magnetic resonance imaging.

Tibiofemoral rotation was measured on axial CT and MRI data as the angle between the tangent of the bony posterior femoral condyles and the tangent of the posterior tibial plateau condyles at the level of the posterior cruciate ligament insertion^12,16^ (Figure 1). Values in which the tibia was externally rotated to the femur were handled as positive values.

**Figure 2.**
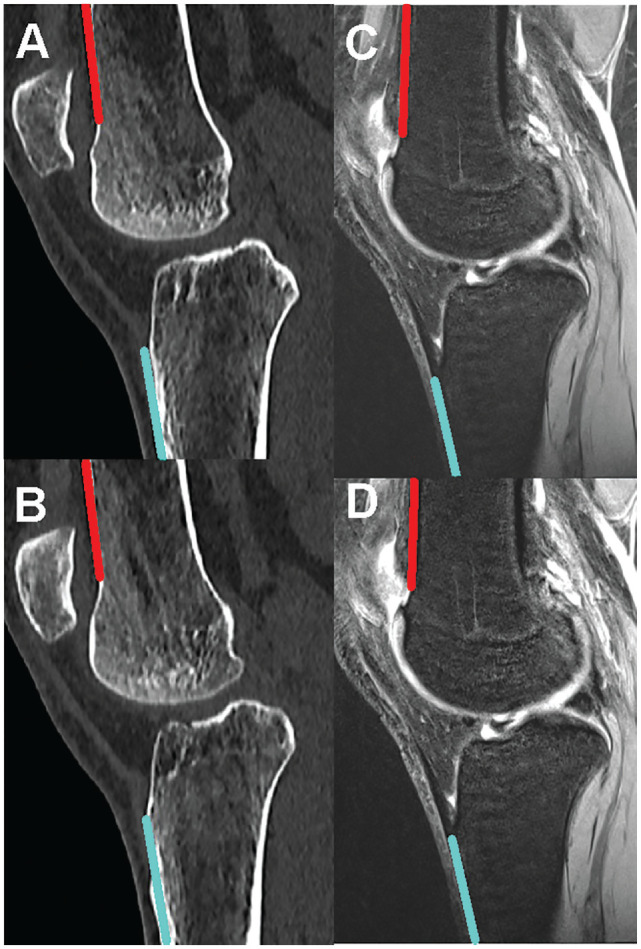
Measurement of the KFA in CT (A and B) and MRI (C and D). The anterior femoral cortex is shown in red (A and C). The anterior tibial cortex is shown in turquoise (B and D). The KFA is measured as the angle between the red and turquoise lines. CT, computed tomography; KFA, knee flexion angle; MRI, magnetic resonance imaging.

The knee flexion angle (KFA) was measured on sagittal CT and MRI data. First, the sagittal image that provided the best depiction of the anterior cortex of the distal femur was identified, which was then marked. Next, the sagittal image on which the anterior cortex of the proximal tibia was best depicted was identified and the anterior cortex was marked. The KFA was then measured as the angle between the 2 lines of the femur and the tibia^19^ (Figure 2).

To ensure interrater reliability, 2 independent readers (A.B. and M.H.), both orthopaedic residents, conducted measurements of tibiofemoral rotation and the KFA in CT and MRI. To ensure intrarater reliability, 1 examiner (A.B.) reassessed the tibiofemoral rotation and the KFA in CT and MRI of 20 randomly selected knees after 2 months to avoid recall bias. All measurements were performed to 1 decimal place.

### Statistical Analysis

Continuous variables are reported as means and standard deviations. The normality of distribution was tested using the Shapiro-Wilk test. Accordingly, the paired *t* test or the Wilcoxon signed-ranked test was applied to assess the differences between CT and MRI measurements. Intraclass correlation coefficients with 95% CIs were calculated for tibiofemoral rotation and KFA measured both on CT and MRI. The association between the KFA and tibiofemoral rotation was evaluated using Pearson correlation and the Mann-Whitney *U* test. All statistical analyses were performed in SPSS for Mac Version 23.0 (SPSS Inc). Significance was set at *P* < .05.

## Results

The patient demographic characteristics are summarized in Table 1.

**Table 1:** Patient Demographic Characteristics^*a*^

No. of patients (No. of knees)	72 (78)
Sex, female/male	49/23
Side, right/left knee	38/40
Age, y	22.2 ± 7.4
BMI, kg/m^2^	25.3 ± 5

a Data presented as n or mean ± SD unless otherwise indicated. BMI, body mass index.

Tibiofemoral rotation measurements were significantly larger in CT than in MRI (*P* < .001), whereas KFA measurements were significantly smaller in CT compared with MRI (*P* < .001). Tibiofemoral rotation measurements in CT and MRI showed a strong correlation (*r* = 0.738; *P* < .001), whereas KFA measurements in CT and MRI showed a weak correlation (*r* = 0.280; *P* = .013). The KFA and tibiofemoral rotation measurements are summarized in Table 2.

**Table 2:** Measurements of Tibiofemoral Rotation and Knee Flexion Angle in CT and MRI^*a*^

	CT	MRI	*P*
Tibiofemoral rotation, deg	8.7 ± 5.5	4.2 ± 6.7	<.001
Knee flexion angle, deg	2.4 ± 3.1	14.9 ± 6.4	<.001

a Data presented as mean ± SD. CT, computed tomography; MRI, magnetic resonance imaging.

The mean difference in tibiofemoral rotation between CT and MRI was 4.6°± 4.6°. The mean difference in the KFA between CT and MRI was 12.6°± 6.3°. The difference in the KFA between CT and MRI (KFA measured in CT – KFA measured in MRI) moderately correlated with the difference in tibiofemoral rotation between CT and MRI (*r* = 0.529; *P* < .001). When comparing CT and MRI measurements, patients with a KFA difference of <10° had a significantly smaller difference in tibiofemoral rotation than patients with a KFA difference of >10° (1.8°± 4.4° versus 6.1°± 3.9°; *P* < .001). According to Landis and Koch,^14^ inter- and intrarater reliability was *almost perfect* for tibiofemoral rotation and the KFA (Table 3).

**Table 3:** Interrater and Intrarater Reliability of Tibiofemoral Rotation and Knee Flexion Angle in CT and MRI^*a*^

	ICC	95% CI
Interrater reliability		
Tibiofemoral rotation CT	0.980	0.940-0.975
Tibiofemoral rotation MRI	0.963	0.942-0.976
Knee flexion angle CT	0.827	0.729-0.890
Knee flexion angle MRI	0.940	0.906-0.962
Intrarater reliability		
Tibiofemoral rotation CT	0.994	0.985-0.998
Tibiofemoral rotation MRI	0.994	0.985-0.998
Knee flexion angle CT	0.983	0.958-0.993
Knee flexion angle MRI	0.977	0.943-0.991

a CT, computed tomography; ICC, intraclass correlation coefficient; MRI, magnetic resonance imaging.

## Discussion

The most important finding of this study was that measurements of tibiofemoral rotation significantly differed between CT and MRI, with larger values observed in CT. Therefore, the hypothesis of this study could be confirmed. Considering this finding, standard values of tibiofemoral rotation, which are inconsistently defined in the literature regarding the used imaging modality, should be considered carefully.

The tibiofemoral rotation represents the relative rotational position of the femur on the tibia, first described in 1997 by Eckhoff et al.^9^ Using CT data, they showed a mean tibiofemoral rotation of 7° in patients with anterior knee pain, compared with 1° in healthy controls. Although tibiofemoral rotation was not widely recognized, it has gained increasing attention in recent years. In 2018, Bernholt et al^4^ showed increased values of tibiofemoral rotation in pediatric and adolescent patients with patellofemoral instability compared with controls without instability. Using MRI data, a mean tibiofemoral rotation of 6.9° in patients with patellofemoral instability and −0.8° in controls was demonstrated. Lin et al^16^ described increased tibiofemoral rotation with increasing clinical severity of patellofemoral instability. A mean tibiofemoral rotation of 8.5° in patients with clinically severe patellofemoral instability, 1.6° in patients with standard traumatic patellofemoral instability, and −3.8° in controls without instability was demonstrated using MRI data. Wu et al^21^ described tibiofemoral rotation in patients with recurrent patellar dislocation using CT data and showed a mean tibiofemoral rotation of 12°, without comparing it with that of healthy controls. However, larger values were observed in their patellofemoral instability group using CT data compared with the previous literature using MRI. Another study by Jud et al^12^ compared tibiofemoral rotation measured in standardized CT data between patients with patellofemoral instability and healthy controls. A mean tibiofemoral rotation of 8.8° for patellofemoral instability and 3.8° for health controls was shown, and larger values were also observed compared with the previous MRI measurements. In addition to the known correlation of increased tibiofemoral rotation and patellofemoral instability, a recent study showed increased odds for failure of ACL reconstruction in patients with a tibiofemoral rotation of ≥4.5° measured using MRI.^15^ Another study by Huettner et al^11^ aimed to investigate normal values of tibiofemoral rotation using MRI data of 100 healthy volunteers. They defined a standard value for tibiofemoral rotation of 1.3° in this healthy population.

The results of this study showed a similar trend to those observed in previous literature, with greater values of tibiofemoral rotation in CT compared with MRI. Significantly different values for tibiofemoral rotation were observed between CT (8.7°± 5.5°) and MRI (4.2°± 6.7°) measurements (*P* < .001), with a mean difference between imaging modalities of 4.6°± 4.6°. The difference in tibiofemoral rotation between CT and MRI moderately correlated with the difference in the KFA between CT and MRI (*r* = 0.529; *P* < .001). The mean difference of the KFA in this study between imaging modalities was 12.6°± 6.3°, with larger remaining flexion in MRI, caused by the required MRI knee coils.^1^ Patients with large KFA differences between CT and MRI in particular showed larger differences in tibiofemoral rotation. Hence, the screw-home mechanism with knee extension may explain the differences in tibiofemoral rotation measurements between imaging modalities. Considering the small differences between normal and pathological values of tibiofemoral rotation described in the literature, the difference in tibiofemoral rotation between imaging modalities seems relevant and pivotal in the judgment if the tibiofemoral rotation of a patient is prone to patellofemoral instability or graft failure after ACL reconstruction. Therefore, a meticulous differentiation of measured tibiofemoral rotation regarding the used imaging modality seems mandatory. The TT-TG distance measurement is a parameter, which is also dependent on the relative position of the femur on the tibia. Therefore, the same characteristic of the dependency on knee flexion and extension was already observed for TT-TG distance measurements.^3,5,7,17^ Likewise, increased values in CT and knee extension, compared with MRI and knee flexion, were described. Meanwhile, these findings lead to a widely applied differentiated consideration of TT-TG between CT and MRI measurements.^2,6,20^

### Limitations

This study should be interpreted in light of its potential limitations. The imaging modalities were standardized only for CT, but MRI was from different institutions using different MRI knee coils. These different knee coils would result in different positions of the joint in terms of knee flexion. However, the position of the respective knee was controlled by the measurement of the KFA. Furthermore, in addition to the patellofemoral instability group, a healthy control group would be favorable. The reason for the lack of a healthy control group was the retrospective nature of this study and the lack of concomitant available CT and MRI scans.

## Conclusion

Tibiofemoral rotation measurements significantly differed between CT and MRI, with larger values observed in CT. The difference between imaging modalities correlated with the degree of knee flexion during image acquisition. This observation should be taken into consideration when assessing tibiofemoral rotation, as current reference values in the literature are inconsistent with regard to the used imaging modality.
